# Case Report: Immediate vs. delayed azithromycin for chronic endometritis: a retrospective cohort study on cure rates and pregnancy outcomes

**DOI:** 10.3389/frph.2025.1721919

**Published:** 2025-12-18

**Authors:** Meng Liu, Xiaoyan Chen, Yaya Wu, Ximin Zhang, Simin Chen, Zhiqiang Liu, Shuyi Yu, Ruochun Lian, Yuye Li

**Affiliations:** 1 Shenzhen Key Laboratory of Reproductive Immunology for Peri-implantation, Shenzhen Zhongshan Institute for Reproductive Medicine and Genetics, Shenzhen Zhongshan Obstetrics & Gynecology Hospital (formerly Shenzhen Zhongshan Urology Hospital), Shenzhen, China; 2 Guangdong Engineering Technology Research Center of Reproductive Immunology for Peri-implantation, Guangzhou, China

**Keywords:** chronic endometritis, CD138, azithromycin, assisted reproductive technology, pregnancy outcomes

## Abstract

**Background:**

Although azithromycin has demonstrated potential therapeutic efficacy in the treatment of chronic endometritis (CE), comprehensive studies on the optimal timing of administration are lacking. Our study aims to evaluate the impact of different timing of azithromycin treatment on cure rates and pregnancy outcomes in patients with CE.

**Methods:**

A retrospective cohort study was conducted involving infertile women diagnosed with CE via hysteroscopy during the proliferative phase. Participants with mild CE were assigned to either: immediate treatment (500 mg oral azithromycin daily for 5 days within the same cycle), or delayed treatment (identical treatment dosage and duration in the subsequent cycle). Follow-up endometrial biopsy with CD138 immunohistochemistry was performed during the secretory phase. Cure rates and pregnancy outcomes were compared between the two groups.

**Results:**

No significant differences in cure rates were observed between the Immediate treatment group (88.40%) and the delayed treatment group (93.63%) (*P* > 0.05). The average time from initial diagnosis to follow-up was significantly shorter in the Immediate treatment group (14.80 ± 3.23 days) compared to the delayed treatment group (44.20 ± 7.00 days) (*P* < 0.0001). Additionally, there were no significant differences in biochemical pregnancy (80.90% vs. 86.39%), clinical pregnancy (70.91% vs. 76.87%), ongoing pregnancy (88.46% vs. 89.38%), or early miscarriage rates (11.54% vs. 8.85%) between the two groups (*P* > 0.05). To further elucidate the relationship between treatment timing and pregnancy outcomes, we performed multivariate regression analysis. This analysis demonstrated that the different treatment timings for CE were not identified as independent risk factors for biochemical pregnancy [1.44 (0.77–2.68), *P* = 0.25], clinical pregnancy [1.35 (0.80–2.28), *P* = 0.264] and ongoing pregnancy [0.83 (0.36–1.88), *P* = 0.65].

**Conclusions:**

In patients with CE, same-cycle treatment offers the advantage of a significantly shorter follow-up time, which may be beneficial for patients undergoing assisted reproductive technology (ART) cycles. Our analysis confirmed that same-cycle treatment significantly accelerates the entire ART process. Furthermore, although the effect of azithromycin treatment timing in chronic endometritis patients did not reach statistical significance, the observed positive trends of pregnant outcomes justify further investigation with larger sample sizes to determine its clinical efficacy.

## Introduction

Chronic endometritis (CE) is a persistent and often asymptomatic inflammatory condition of the endometrium, characterized by the infiltration of plasma cells into the endometrial stroma (1). CE can impair endometrial receptivity (2) and disrupt the local immune microenvironment of the endometrium (3), making it an important etiological factor in infertility and adverse pregnancy outcomes (4, 5). Numerous studies have demonstrated a strong association between CE and infertility, repeated implantation failure (RIF), or recurrent pregnancy loss (RPL) (4, 6, 7). According to the reports the incidence of CE among infertile women ranges from 0.2% to 46% (6, 8, 9), and is as high as 67.6% in women with RIF (10) and 56.8% in women with RPL respectively (11).

Currently, immunohistochemical detection of CD138+ plasma cells in the endometrium is considered a more accurate diagnostic method for CE (12). However, the lack of standardized diagnostic criteria and inconsistencies in sampling timing among most laboratories contribute to the wide variation in reported prevalence of CE. Our previous research demonstrated that the number of CD138+ plasma cells in the proliferative phase is significantly higher than in the secretory phase, prompting the establishment of phase-specific diagnostic criteria for CE based on CD138+ cell counts in the proliferative and secretory phases (13, 14).

The primary cause of CE is intrauterine microbial infection triggered by a wide range of microorganisms, including common bacteria (e.g., *Escherichia coli*, *Enterococcus faecalis*, *Streptococcus*, and *Staphylococcus*), *Mycoplasma*, *Ureaplasma*, and *Mycobacterium* species (15–17). A meta-analysis confirmed that oral antibiotic therapy significantly improves CE resolution in most patients and enhances subsequent IVF outcomes (18). Among the commonly used antibiotics for CE, doxycycline is one of the most frequently prescribed (19, 20). Doxycycline typically requires a 14-day course of multiple doses (21). Other antibiotics reported for CE treatment include metronidazole, levofloxacin (22), and azithromycin (10). Azithromycin is an acid-stable macrolide antibiotic structurally, with a broad antimicrobial spectrum (23). It inhibits bacterial protein synthesis by targeting the 50S ribosomal subunit and is effective against *Chlamydia trachomatis*, *Neisseria gonorrhoeae*, *Mycoplasma/Ureaplasma*, and certain anaerobic bacteria (24). It is widely used in the treatment of sexually transmitted infections (21).Studies have shown that azithromycin is more effective than doxycycline in curing mild to moderate pelvic inflammatory disease (PID) (25). However, azithromycin is rarely used in the treatment of CE. Compared with other antibiotics such as doxycycline, azithromycin offers a shorter treatment duration and lower dosing frequency (26). This shorter treatment period is a major advantage, as patient adherence is generally inversely proportional to the length of the regimen. More importantly, azithromycin exhibits high tissue bioavailability and strong tissue penetration, with concentrations in macrophages and inflamed tissues reaching 50–100 times those in serum. Its tissue half-life ranges from 2 to 4 days (27). These pharmacokinetic properties allow for a significantly shortened dosing schedule while maintaining high antibacterial activity at the cellular level and within specific tissue compartments.

However, standardized treatment protocols for azithromycin in CE remain lacking, and no systematic studies have been conducted on the optimal timing of treatment. Employing a retrospective cohort design, this study evaluated the impact of a 7-day oral azithromycin treatment—timed either in the same menstrual cycle or the following cycle—on cure rates and pregnancy outcomes among patients with mild chronic endometritis. The findings aim to provide evidence for clinical guidance in CE management.

## Materials and methods

### Subjects

This retrospective study was approved by the Ethics Committee of Shenzhen Zhongshan Obstetrics and Gynecology Hospital (Approval No. SZZSECHU-2024085) and all participants provided written informed consent. All procedures were conducted in accordance with the Declaration of Helsinki (as revised in 2013).

**Figure 1:**
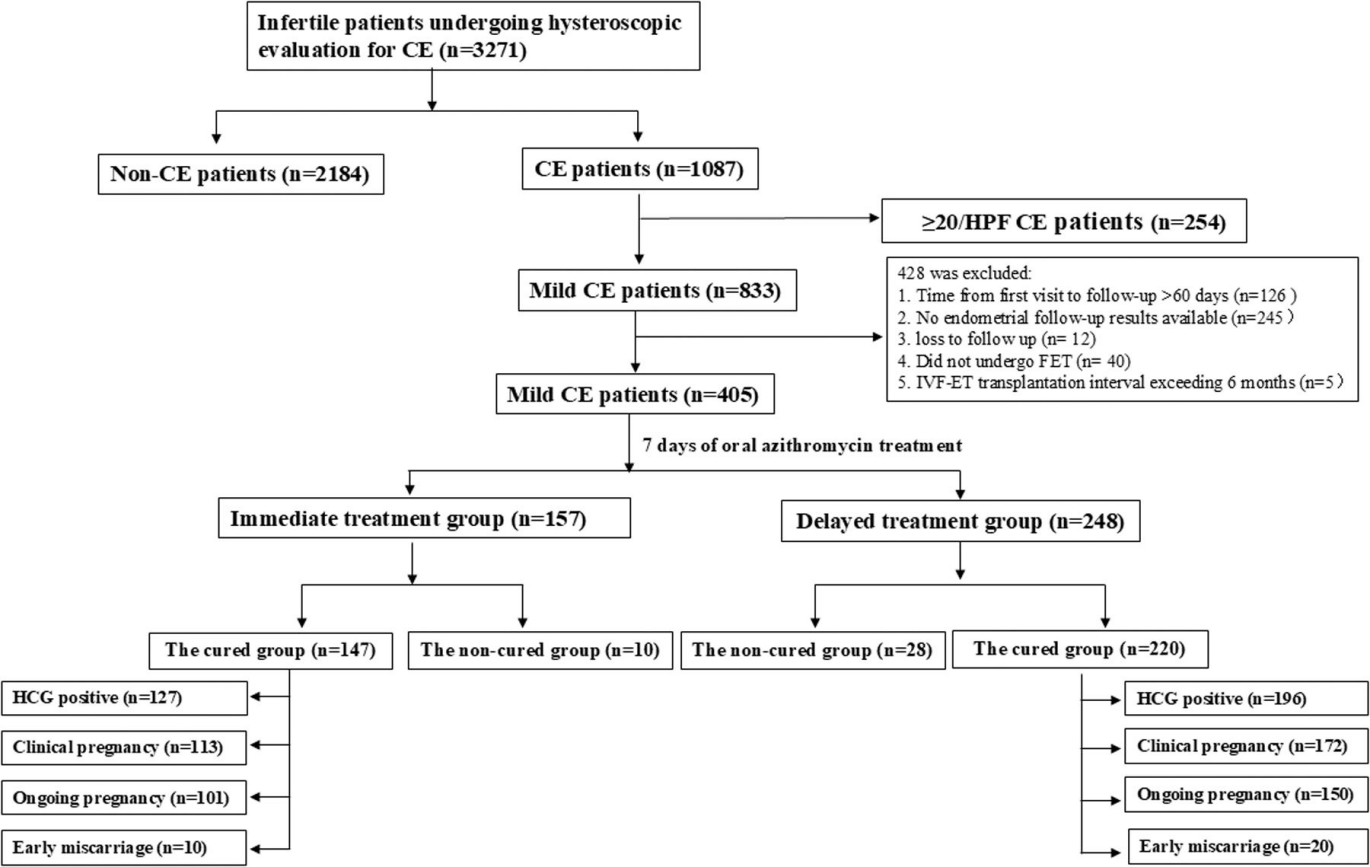
Flowchart of patient inclusion. A total of 3,271 infertile patients who visited the Fertility Center and underwent hysteroscopic evaluation for CE were enrolled in this study. Based on the criteria, 20 patients were excluded. All remaining 405 mild CE patients and 367 cured CE patients were followed-up to determine the pregnancy outcome.

A retrospective analysis was conducted through the hospital's electronic medical record system on infertile patients who underwent hysteroscopic examination during the proliferative phase and were screened for chronic endometritis at Shenzhen Zhongshan Maternity & Child Healthcare Hospital from January 2022 to August 2024 (Figure 1). Inclusion criteria were as follows: Diagnosed with chronic endometritis (CE); CD138+ plasma cells ≤ 20 per high-power field (HPF, mild CE); Post-hysteroscopy transvaginal ultrasound indicating endometrial thickness ≥ 7 mm. Patients with intrauterine adhesions, thin endometrium (endometrial thickness < 7 mm), or suspected endometrial hyperplasia/malignancy were excluded from the study.

**Figure 2:**
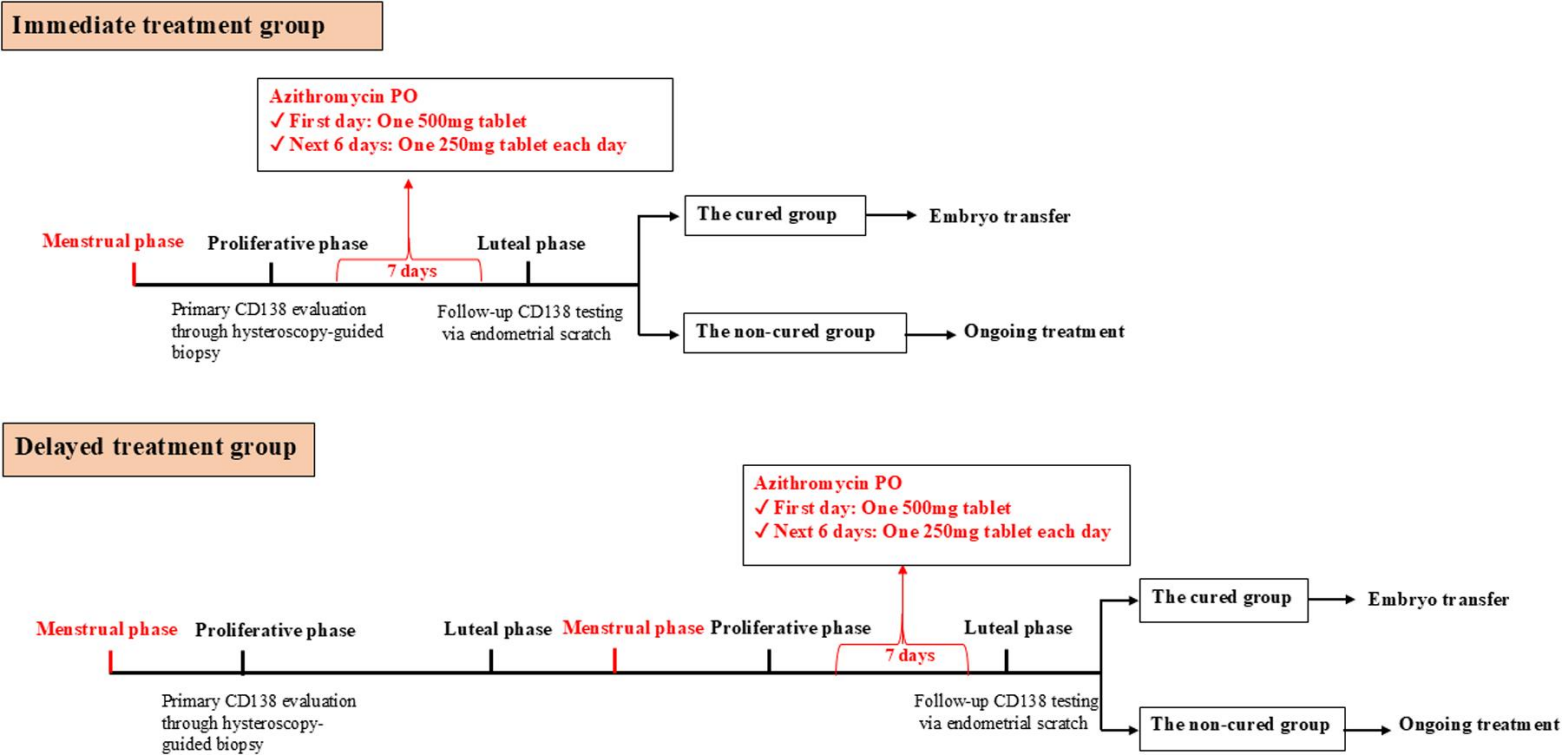
Flowchart of treatment and follow-up in the immediate treatment group and delayed treatment group. The Immediate treatment group is defined as patients underwent hysteroscopic evaluation followed by a 7-day course of oral azithromycin (500 mg on day 1, 250 mg daily on days 2–6) and CD138 re-assessment within the same menstrual cycle. The Delayed treatment group is defined as Patients received the same treatment regimen in the subsequent menstrual cycle following hysteroscopy, with CD138 re-evaluation conducted in that cycle.

Based on the timing of treatment, patients were divided into two groups:

Following a confirmed diagnosis of chronic endometritis by CD138 immunohistochemical staining, patients in the immediate-treatment group commenced oral azithromycin (500 mg on day 1, followed by 250 mg daily for 6 days) during the proliferative phase of the same diagnostic cycle. In contrast, patients in the delayed-treatment group initiated the identical azithromycin regimen during the proliferative phase of the subsequent menstrual cycle. Therapeutic efficacy was evaluated by a follow-up endometrial scratching and CD138 testing performed approximately 7 days after the completion of treatment (i.e., in the luteal phase of the respective cycle). Patients who achieved cure subsequently proceeded to frozen-thawed embryo transfer (Figure 2).

Cured group was defined as the resolution of chronic endometritis following azithromycin treatment, evidenced by a reduction in CD138+ plasma cell counts from ≥5/HPF to <5/HPF or zero. Non-cured group was defined as the persistence of chronic endometritis after azithromycin treatment, with CD138+ plasma cell counts remaining at ≥5/HPF.

### Hysteroscopy

Hysteroscopic examination was performed 3 to 7 days after the end of menstruation. Using a 3.1 mm-diameter flexible hysteroscope with a continuous flow system (Olympus, Tokyo, Japan), the procedure was conducted during the proliferative phase (days 6–12 of the menstrual cycle). Endometrial biopsy samples were obtained using a 3-mm-wide curette (Atom Medical, Tokyo, Japan).

Normal saline was used as the distension medium. During anesthesia, the initial intrauterine pressure was set at 60–80 mmHg and adjusted intraoperatively as needed to achieve adequate uterine distension and ensure a clear visual field. Endometrial biopsy was performed in all patients. Specimens were fixed in formalin and sent to the pathology department for immunohistochemical staining.

### Endometrial biopsy

Endometrial biopsies during the secretory phase were obtained using a standard technique with a Pipelle catheter (Jiangxi Nuode Medical Healthy Science Company, Jiangxi, China). The collected specimens were immediately placed in 10% neutral buffered formalin and fixed at room temperature for 4–6 h. Following fixation, the tissues were dehydrated and embedded in paraffin.

### Immunohistochemical staining

Paraffin-embedded endometrial tissues were sectioned into 4 μm slices, deparaffinized in xylene, and rehydrated through a graded alcohol series. Immunohistochemistry (IHC) staining was performed using a Leica Bond III automated immunostainer (Leica Biosystems, Wetzlar, Germany) and the Bond Polymer Refine Detection kit (DS9800, Leica Microsystems, Wetzlar, Germany) according to the manufacturer's instructions. Briefly, antigen retrieval was conducted by heating the slides for 20 min at 100°C in an ethylenediaminetetraacetic acid (EDTA) solution. Endogenous peroxidase activity was blocked using 3% hydrogen peroxide (H₂O₂) in methanol for 10 min. The slides were then incubated with a CD138 primary antibody (1:250 dilution; Gene Tech, Shanghai, China) for 30 min, followed by a 30-minute incubation with a horseradish peroxidase–conjugated secondary antibody. Immunoreactivity was visualized using 3,3′-diaminobenzidine (DAB) as the chromogen, and hematoxylin was used for counterstaining. A Tissue-Tek Film Automated Coverslipper (Sakura Finetek, California, USA) was used for slide mounting.

### Identification and quantification of CD138^+^ plasma cells

Chronic endometritis was diagnosed by counting CD138^+^ plasma cells within the endometrial stroma under a light microscope (Nikon, Melville, New York) at 200× magnification (0.0625 mm^2^ per field). CD138^+^ cells were identified as plasma cells with strong membranous immunopositivity, weak cytoplasmic staining, and a round nucleus eccentrically located with a characteristic “spoke-wheel” chromatin pattern along the nuclear periphery.

CD138^+^ cell counts were assessed independently by two experience pathologists. Any discrepancies were resolved through discussion or, when necessary, through consensus with a senior pathologist.

Since diagnostic criteria for chronic endometritis (CE) vary depending on the phase of the menstrual cycle during which the endometrial biopsy is performed, this study adopted the following standards: For secretory phase endometrium, CE was diagnosed when at least one positive focus was observed in the endometrial stroma (13). For proliferative phase endometrium, CE was diagnosed when at least three positive foci were identified in the endometrial stroma, with one positive focus defined as ≥5 CD138^+^ plasma cells per high-power field (HPF) (14). The diagnosis of mild CE was established when the positive foci contained 5–20 CD138^+^ cells per foci.

### Azithromycin treatment

Patients who met the diagnostic criteria for proliferative phase endometrial CE and the inclusion criteria were enrolled. In the Immediate treatment group, azithromycin was administered orally (500 mg on the first day, 250 mg on the second day), once daily for 7 consecutive days, starting after the hysteroscopy. Endometrial tissue samples were collected during the secretory phase of the same cycle. In the Delayed treatment group, azithromycin was administered orally (500 mg on the first day, 250 mg on the second day), once daily for 7 consecutive days, starting on the first day of menstruation in the following cycle. Endometrial tissue samples were collected during the secretory phase of the same cycle as the hysteroscopy (Figure 2).

### *In vitro* fertilization-embryo transfer (IVF-ET)

All the infertile patients enrolled underwent frozen embryo transfer through *in vitro* Fertilization-Embryo Transfer (IVF-ET).

All patients underwent controlled ovarian stimulation. When at least three dominant follicles reached a mean diameter of 18 mm, human chorionic gonadotropin (hCG) was administered to trigger final oocyte maturation. Transvaginal ultrasound-guided oocyte retrieval was performed 36 h later. The retrieved oocytes were fertilized by intracytoplasmic sperm injection (ICSI), and the resulting good-quality blastocysts were cryopreserved using vitrification.For the frozen-thawed embryo transfer (FET) cycles, the endometrium was prepared using a hormone replacement therapy (HRT) protocol. Exogenous progesterone supplementation was initiated when the endometrial thickness reached ≥8 mm. The first day of progesterone administration was defined as the luteal conversion day, representing the starting day of endometrial transformation from the proliferative phase to the secretory phase. The frozen blastocysts were thawed and transferred on the fifth day following luteal conversion.

### Pregnancy outcomes measurement

The primary outcome was live birth after embryo transfer, defined as the delivery of a baby following a successful pregnancy. Secondary outcomes included the following:

Biochemical Pregnancy: a serum hCG level exceeding 10 U/L, measured 14 days after embryo transfer; Clinical Pregnancy: a clinical pregnancy is defined as the presence of a gestational sac on ultrasound at seven to eight weeks of gestation; Ongoing Pregnancy: an ongoing pregnancy is defined as the detection of at least one fetal heartbeat on ultrasound beyond 12 weeks of gestation; Early miscarriage: the spontaneous pregnancy loss occurring within the first 12 weeks of gestation.

### Statistical analysis

Data were presented as means ± standard deviation (SD) for normally distributed data, medians with interquartile ranges (IQRs) for non-normally distributed data, and frequencies (percentages) for categorical variables. Nonparametric t-tests were used for intergroup comparisons, with the Kruskal–Wallis test applied to continuous variables and Pearson Chi-square test for categorical variables. The prognostic significance of azithromycin treatment at different treatment timings for pregnancy outcomes was assessed using multivariable logistic regression models. Potential confounders such as age, body mass index (BMI), anti-Müllerian hormone (AMH) levels, infertility duration and type, embryo characteristics (e.g., number of transferred embryos, embryo quality, type, and method of embryo transfer), and fertilization methods were adjusted for. All statistical analyses were performed using SPSS Statistics version 20.0 (SPSS Inc., Chicago, IL, USA). A *P* ≤ 0.05 was considered statistically significant.

## Results

### Clinical characteristics of infertile patients

A total of 3,271 infertile patients underwent hysteroscopy for chronic endometritis (CE) screening, of which 1,087 patients were diagnosed with CE (Figure 1), resulting in a prevalence of approximately 33.23% (1,087/3,271). 833 CE patients were mild CE and according the inclusion and exclusion criteria, 405 mild CE patients were included in the retrospective analysis. These patients were divided into two groups based on the timing of treatment: Immediate treatment group (*n* = 248) and Delayed treatment group (*n* = 157, Figure 2). A comparison of the clinical baseline information between the two groups revealed that most of the basic characteristics, including patient age, number of pregnancies, infertility type, causes of infertility, number of transferred high-quality embryos, and embryo characteristicswere similar (Table 1). However, compared with the Delayed treatment group, the Immediate treatment group had a higher BMI (22.32 ± 3.31 vs. 21.38 ± 2.92, *P* = 0.004), higher AMH levels (4.14 ± 2.72 vs. 3.60 ± 2.64, *P* = 0.026), and relatively thicker endometrial thickness on the secretory phase conversion day (9.96 ± 1.69 vs. 9.48 ± 1.78, *P* = 0.005).

**Table 1:** Baseline clinical characteristics of patients.

Variable	Immediate treatment group (*N* = 220)	Delayed treatment group (*N* = 147)	*P*-value
Age (years)	33.64 ± 4.89	33.09 ± 4.40	0.498
BMI (kg/m^2^)	22.32 ± 3.31	21.38 ± 2.92	0.004
Parity (times)	0.25 ± 0.53	0.25 ± 0.54	0.913
Duration of infertility (years)	3.44 ± 2.82	3.25 ± 2.77	0.489
Type of infertility			0.580
Primary	99/220 (45.00%)	71/147 (48.30%)	
Secondary	121/220 (55.00%)	76/147 (51.70%)	
Cause of infertility			0.432
Unexplained	50/220 (22.73%)	24/147 (16.33%)	
Male factor	31/220 (14.09%)	45/147 (30.61%)	
Pelvic and tubal factors	114/220 (51.82%)	87/147 (59.18%)	
Mix factor	35/220 (15.91%)	20/147 (13.61%)	
Endometriosis	25/220 (11.36%)	16/147 (10.88%)	
AMH (ng/mL)	4.14 ± 2.72	3.60 ± 2.64	0.026
Endometrial thickness at progesterone conversion	9.96 ± 1.69	9.48 ± 1.78	0.005
No. of transferred high-quality embryos	1.05 ± 0.63	1.03 ± 0.65	0.766
Embryo type			0.822
Cleavage	44/245 (17.96%)	28/147 (19.05%)	
Blastocyst	176/245 (71.84%)	119/147 (80.95%)	

Mean ± SD; *n*/*N*(%), Welch Two Sample *t*-test; Pearson's Chi-squared test; Fisher's exact test.

### The 7-day oral azithromycin treatment does not affect the cure rate of mild CE based on different treatment timings

To investigate whether the timing of treatment influences the cure rate of CE, we further analyzed the results of the follow-up for CE patients. The results showed that in the Immediate treatment group, 220 patients were cured, with a cure rate of 88.40% (220/248). In the Delayed treatment group, 147 patients were cured, with a cure rate of 93.63% (147/157). There was no significant difference in the CE cure rates between the two groups (Table 2, *P* = 0.098), suggesting that 7-day oral azithromycin treatment in the same month do not affect the cure rate of CE.

**Table 2:** Impact of different follow-up timing on cure rates in CE.

Comparison of cure rates and follow-up duration between immediate and delayed treatment groups in CE patients	Immediate treatment group	Delayed treatment group	*P*-value
Cure rate of CE (%)	220/248 (91.67%)	147/157 (93.63%)	0.098
Mean duration from initial visit to follow-up (days)	14.80 ± 3.23	44.20 ± 7.00	0.000

Additionally, the average time from initial diagnosis to follow-up was compared between the two groups. The Immediate treatment group had a significantly shorter average follow-up time (14.80 ± 3.23 vs. 44.20 ± 7.00, *P* < 0.000), with an average reduction of 30 days. Compared to the delayed treatment which inherently requires a longer waiting period, the immediate treatment integrates diagnosis, treatment, and evaluation into a single cycle. This consolidation significantly reduces the total treatment duration, allowing patients to proceed to embryo transfer sooner.

### The 7-day oral azithromycin treatment at different treatment timings does not significantly affect pregnancy outcomes in patients with mild CE

To further clarify whether the timing of treatment affects pregnancy outcomes in patients cured of CE, we conducted a detailed analysis of pregnancy outcomes and used multivariable logistic regression to identify independent risk factors for pregnancy outcomes. As shown in Table 3, the results revealed that compared to the Delayed treatment group, the same-month group had similar biochemical pregnancy rate (80.90% vs. 86.39%, *P* = 0.169), clinical pregnancy rate (70.91% vs. 76.87%, *P* = 0.206), ongoing pregnancy rate (88.46% vs. 89.38%, *P* = 0.813), and early miscarriage rate (11.54% vs. 8.85%, *P* = 0.476), all of which were not statistically significant (Table 3).

**Table 3:** Impact of different follow-up timing on pregnancy outcomes.

Pregnancy outcomes	Immediate treatment group (*N* = 220)	Delayed treatment group (*N* = 147)	*P*-value
Biochemical pregnancy			0.169
Positive	178/220 (80.90%)	127/147 (86.39%)	
Negative	42/220 (19.09%)	20/147 (13.61%)	
Clinical pregnancy			0.206
Positive	156/220 (70.91%)	113/147 (76.87%)	
Negative	64/220 (20.09%)	34/147 (23.13%)	
Ongoing pregnancy			0.813
Yes	138/156 (88.46%)	101/113 (89.38%)	
No	18/156 (11.54%)	12/113 (10.62%)	
Early miscarriage			0.476
Yes	18/156 (11.54%)	10/113 (8.85%)	
No	138/156 (88.46%)	103/113 (91.15%)	

After adjusting for age, BMI, duration of infertility, AMH, number of transferred high-quality embryos, and embryo attributes using multivariable logistic regression, we found that age was an independent factor influencing biochemical pregnancy [1.10 (1.03–1.18), *P* = 0.007] and clinical pregnancy [1.13 (1.06–1.20), *P* < 0.000]. However, the different treatment timings for CE were not identified as independent risk factors for biochemical pregnancy [1.44 (0.77–2.68), *P* = 0.25], clinical pregnancy [1.35 (0.80–2.28), *P* = 0.264] and ongoing pregnancy [0.83 (0.36–1.88), *P* = 0.65] (Table 4).

**Table 4:** Logistic regression analysis on the contribution of medication follow-up timing to pregnancy outcome.

Variable	Biochemical pregnancy (*N* = 367)		Clinical pregnancy (*N* = 367)		Ongoing pregnancy (*N* = 269)		Early miscarriage (*N* = 269)	
	aOR (95% CI)	*P*	aOR (95% CI)	*P*	aOR (95% CI)	*P*	aOR (95% CI)	*P*
Medication follow-up timing	1.44 (0.77–2.68)	0.25	1.35 (0.80, 2.28	0.264	0.83 (0.36, 1.88)	0.65	0.98 (0.42, 2.33)	0.969
Age (years)	1.100 (1.03–1.18)	0.007	1.13 (1.06,1.20)	0.000	1.01 (0.92, 1.11)	0.82	0.99 (0.89, 1.09)	0.76
BMI (kg/m^2^)	1.00 (0.92–1.10)	0.94	0.99 (0.91, 1.07)	0.761	1.10 (0.98, 1.23)	0.10	0.92 (0.82, 1.03	0.16
Duration of infertility (years)	1.10 (1.00–1.20)	0.05	1.03 (0.95, 1.13)	0.48	1.07 (0.94, 1.21	0.30	0.92 (0.81, 1.05)	0.23
AMH (ng/mL)	1.02 (0.90–1.15)	0.80	1.08 (0.98, 1.19)	0.13	1.15 (0.98, 1.34)	0.09	0.86 (0.68, 1.09)	0.07
Endometrial thickness at progesterone conversion	0.90 (0.75, 1.07)	0.22	0.86 (0.73, 1.00)	0.049	1.14 (0.91, 1.43)	0.26	0.86 (0.68, 1.09)	0.22
Number of high-quality embryos transferred	0.99 (0.55–1.78)	0.96	0.76 (0.46, 1,27))	0.29	0.83 (0.37 1.86)	0.65	1.30 (0.56, 2,97)	0.55
Embryo type (blastocyst)	0.49 (0.19–1.28)	0.15	0.31 (0.13, 0.70	0.005	0.28 (0.08, 1.07)	0.06	3.45 (0.88, 13.62)	0.08

OR, odds ratio; CI, confidence interval; aOR, adjusted odds ratio; AMH, anti-Müllerian hormone.

## Discussion

This study is the first to systematically explore the impact of follow-up timing after 7 days of oral azithromycin treatment for mild CE. Our results indicate that there is no significant difference in cure rates and pregnancy outcomes between patients who underwent follow-up in the same cycle (the Immediate treatment group) vs. those who received follow-up in the subsequent cycle (the Delayed treatment group). These findings suggest that the timing of follow-up after azithromycin treatment does not influence the overall treatment success or pregnancy outcomes for mild CE patients.

Currently, there is no standardized antibiotic treatment of CE. Cicinelli et al. proposed a stratified antibiotic approach based on the uterine microbial profile in women with recurrent implantation failure and CE-related infertility (8). However, due to the challenges in the identification and culture of bacterial, most studies report empirical antibiotic use for CE treatment. Because their broad-spectrum antimicrobial activity against both common bacteria and mycoplasma, tetracycline, metronidazole, and levofloxacin have been frequently used in the management of CE (28). Johnson-MacAnanny et al. administered oral doxycycline (100 mg, twice daily for 14 days) to women with recurrent implantation failure and CE. They found that 70% (7/10) of the women cleared CD138^+^ plasma cells in the endometrial stroma. The remaining three patients required additional treatment with ciprofloxacin and metronidazole (500 mg, twice daily for 14 days) to eradicate the endometrial plasma cells (19). McQueen et al. reported that for women with recurrent miscarriage and CE, a combination of ofloxacin (400 mg, twice daily for 14 days) and metronidazole (500 mg, twice daily for 14 days) resulted in a histopathological cure rate of 73% (19/26) (29). However, there are relatively few reports on azithromycin treatment of CE. Azithromycin, as an acid-stable macrolide with strong tissue penetration and relatively short course of administration, has shown promising efficacy in treating various infectious diseases, including PID (30). In this study, we observed a nearly 90% cure rate for mild CE patients, which is significantly higher than previously reported conversion rates, possibly due to stricter diagnostic criteria in our center (13, 14) and the relatively lower inflammatory burden in mild cases. These findings are consistent with previous studies showing that azithromycin is an effective antibiotic for treating CE, as it was comparable in efficacy to other treatments, such as moxifloxacin, in managing multidrug-resistant forms of the disease (31).

Emerging evidence highlights a significant association between endometriosis and CE, with both conditions sharing common pathological features such as aberrant immune cell infiltration and a proinflammatory microenvironment (32–34). In our study, the prevalence of endometriosis was comparable between the Immediate and Delayed treatment groups. This suggests that the efficacy of follow-up is consistent regardless of endometriosis status, further supporting the robustness of our findings across different patient profiles.

There are few studies reporting on the timing of follow-up after chronic endometritis treatment. Song D et al. mentioned that women with CE who received antibiotic treatment (levofloxacin 500 mg and tinidazole 1,000 mg daily for 14 days) underwent repeat hysteroscopy and biopsy for histopathological CD138 immunohistochemical testing 2–4 weeks after antibiotic treatment to confirm whether CE was still present (35).Other studies did not clearly describe the timing of the second biopsy follow-up, such as Qingyan Zhang et al., who mentioned that women diagnosed with chronic endometritis through hysteroscopy and biopsy received standard antibiotic treatment, and the second biopsy confirmed the resolution of CE (36). Many studies did not perform CD138 follow-up biopsy after antibiotic treatment for CE (37). In our study found that in the same-month treatment group, endometrial scraping during the secretory phase followed by frozen embryo transfer in the next month resulted in a significant reduction in waiting time (average shortening by 30 days). This markedly reduced the waiting time for embryo transfer in assisted reproduction patients and improved the benefits for IVF patients. For patients planning frozen embryo transfer, we recommend the same-month treatment and next-month transfer protocol, which can avoid the two-month wait in traditional protocols, especially for older or ovarian function diminished patients.It is also worth noting that while the overall cure rate and pregnancy outcomes did not differ between the two groups, this study did not assess long-term pregnancy outcomes such as live birth rates or perinatal complications. Further studies are warranted to explore these outcomes, as well as the potential long-term effects of azithromycin treatment on reproductive health.

Our study demonstrated adjusted odds ratios (aORs) >1.25 for both biochemical and clinical pregnancy in the same-month treatment group, suggesting a potential positive effect of the intervention on pregnancy outcomes. However, the wide 95% confidence intervals and lack of traditional statistical significance (*P* > 0.05) indicate that these findings should be interpreted cautiously. The absence of significance likely stems from limited statistical power due to the small sample size, which increases standard errors, widens confidence intervals, and reduces precision in estimating the true effect. Additionally, the small-sample design introduces risks of bias, including baseline population heterogeneity (e.g., variations in age, comorbidities, and prior pregnancy history) and potential residual confounding from inadequately balanced factors.

A number of limitations in this study should be considered. First, its retrospective design may have introduced selection bias, particularly as only patients with mild CE were included. Second, the absence of a comparator group (e.g., doxycycline treatment or a no-treatment control) limits the ability to assess the relative efficacy of the azithromycin protocol or control for spontaneous resolution. Furthermore, the single-center nature of the sample limits generalizability, as the findings may not reflect outcomes in more diverse populations. While our data support the feasibility of same-month follow-up, these results require validation through larger, prospective randomized controlled trials. Standardization of CE diagnostic criteria across menstrual cycles (based on hysteroscopy and biopsy) is also needed to enhance diagnostic accuracy. Future research should prioritize confirming the long-term benefits of this intervention and evaluating its applicability to broader patient subgroups.

In summary, this study provides evidence that same-month follow-up after azithromycin treatment is as effective as the conventional next-month approach in assessing treatment response in patients with mild cystic endometritis. These findings support a more time-efficient management strategy, particularly for patients undergoing ART, as it enables earlier treatment decisions without sacrificing therapeutic efficacy or endometrial receptivity. This has important implications for optimizing antimicrobial treatment protocols in reproductive medicine.

## Data Availability

The raw data supporting the conclusions of this article will be made available by the authors, without undue reservation.
